# *Lactobacillus plantarum* GKM3 Promotes Longevity, Memory Retention, and Reduces Brain Oxidation Stress in SAMP8 Mice

**DOI:** 10.3390/nu13082860

**Published:** 2021-08-20

**Authors:** Shih-Wei Lin, You-Shan Tsai, Yen-Lien Chen, Ming-Fu Wang, Chin-Chu Chen, Wen-Hsin Lin, Tony J. Fang

**Affiliations:** 1 Department of Food Science and Biotechnology, National Chung Hsing University, Taichung 402204, Taiwan; wei.lin@grapeking.com.tw; 2 Biotech Research Institute, Grape King Bio Ltd., Taoyuan 325002, Taiwan; you.shan.tsai@gmail.com (Y.-S.T.); lan.chen@grapeking.com.tw (Y.-L.C.); 3 Department of Food and Nutrition, Providence University, Taichung 433303, Taiwan; mfwang@pu.edu.tw; 4 Institute of Food Science and Technology, National Taiwan University, Taipei 106319, Taiwan; gkbioeng@grapeking.com.tw; 5 Department of Food Science, Nutrition and Nutraceutical Biotechnology, Shih Chien University, Taipei 104336, Taiwan; 6 Department of Bioscience Technology, Chung Yuan Christian University, Taoyuan 320314, Taiwan; 7 Department of Pharmacy, China Medical University, Taichung 404333, Taiwan

**Keywords:** senescence accelerated mouse prone 8 (SAMP8), probiotics, 8-hydroxy-2′-deoxyguanosine (8-OHdG), thiobarbituric acid-reactive substances (TBARS), amyloid-β (Aβ), aging

## Abstract

(1) Background: An age-related cognitive decline is commonly affecting the life of elderly with symptoms involved in progressive impairments to memory and learning. It has been proposed that probiotics could modulate age-related neurological disorders via the gut–brain axis. (2) Methods: To investigate the anti-aging effect of probiotic *Lactobacillus plantarum* GKM3, both survival tests and cognitive experiments were conducted in the SAMP8 mice model. The six-month-old SAMP8 (n = 20 in each gender) were fed with probiotic GKM3 at a dosage of 5.1 × 10^9^ and 1.0 × 10^9^ cfu/ kg B.W./day until their natural death. Then, the life span was investigated. Three-month-old SAMP8 (n = 10 in each gender) were administered GKM3 for 14 weeks. Then, the behavior tests and oxidation parameters were recorded. (3) Results: GKM3 groups showed significantly increased latency in the passive avoidance test and time of successful avoidance in the active avoidance test. The TBARS and 8-OHdG from mice brains also showed a significant reduction in the groups treated with GKM3. In addition, lower accumulation of the amyloid-β protein was found in SAMP8 mice brains with the supplement of GKM3. (4) Conclusions: These results indicated that *L. plantarum* GKM3 delayed the process of aging, alleviated age-related cognitive impairment, and reduced oxidative stress.

## 1. Introduction

It has been an issue that population-suffered cognitive impairment has become bigger following the growth of the amount of older individuals in the last decade [1]. According to the report from the World Health Organization (WHO), the number of people with cognitive decline, which was more than 46.8 million in 2015, will reach over 74.7 million in 2030, which is almost increased by half within fifteen years [2]. Aging, recognized as a strong correlation with cognitive impairment, is generally thought to be associated with oxidative stress, which is involved in the accumulation of free radicals and promoted many physiological dysfunctions especially in the brain, an oxygen-sensitive organ, leading to degenerative symptoms such as memory loss and decline of learning ability [3,4,5].

As aging is inevitable and its relative negative symptoms are complicated, people pursue treatments for anti-aging, ranging from diet therapies to more than drug treatments [6,7]. For example, ginseng (*Panax quinquefolius*) root extract demonstrated improved cognitive function and was marked as a favorite by the Koreans [8]. *Ginkgo biloba* extract, another example, was loved by the Japanese for its functions in cognitive enhancing and oxidation-reduction [9]. Although many reports have suggested that the plant-based diets provide antioxidant effects and retard the aging process, there are more interesting focuses on the gut microbiota or even on a group of specific bacteria in the gut that could metabolize these nutraceuticals and provide positive multi-modulation effects to the host, including longevity [10,11,12,13,14]. Gut bacteria could produce short-chain fatty acids (SCFAs) such as acetate, propionate, or butyrate from one’s diet [15]. Children who consumed plant-based enteral nutrition showed higher SCFAs than the normal kids in their stool samples [16]. These SCFAs can play an important role in the human gastrointestinal epithelium [17]. Acetate can cross the blood-brain barrier, inducing hypothalamic neuronal activation [18]. In addition, butyrate has been studied regarding the effect of suppressing colonic inflammation in the human gut [19]. Therefore, it matters what kind of microbiota or specific bacteria is in the human gut, especially in terms of influencing body health and longevity [20,21].

The intestinal microbiota also can impact the gut-brain axis and delay the process of aging, thereby preventing the progress of age-related diseases such as amnesia, dementia, or even Alzheimer’s disease [22,23]. The neurotransmitter gamma-aminobutyric acid (GABA), for example, could be produced by certain bacteria strains that have been reported with the proliferation of epithelial stem cells in the gut and the improvement of depressive-like behavior in mice [24,25]. With current supportive data, the addition of probiotics as a prevention method has been a trend for these age-related neurological disorders [26,27].

*Lactobacillus* is the most common probiotic that showed a high possibility of developing functional food as it has existed in human food for a long time [28]. However, individual bacterial species have unique bioactivities, from strains to strains, that require experimental confirmation. In our previous studies, we isolated the probiotic strain *Lactobacillus plantarum* GKM3 from pickled leaf (*Brassica juncea*), also known as the longevity vegetable in Taiwanese Hakka society, and conducted functional studies on boosting the gastrointestinal tract and immunity in vivo [29,30,31]. Therefore, we expected the likelihood of *L. plantarum* GKM3 alleviating the process of aging and age-related cognitive impairment.

## 2. Materials and Methods

### 2.1. Subjects

*L. plantarum* GKM3 was isolated from Chinese mustard (*Brassica juncea*) and preserved at the Bioresource Collection and Research Center, Taiwan (BCRC), with the collection number 910787. First, GKM3 was cultured with a MRS (BD Co., Franklin Lakes, NJ, USA) broth at 37 °C for 18 h in the test tube. With 0.01% inoculation, the GKM3 seed culture was scaled up in 50 L fermenter under a pH 6.0 control at 37 °C for 18 h with a designed medium (5% glucose, 2% yeast extract, 0.05% MgSO_4_, 0.1% K_2_HPO_4_, and 0.1% Tween 80). Then, the fermented bacteria were harvested by centrifugation at 3800 rpm for 10 min, washed with phosphate saline buffer twice, and the pellet was mixed with 10% milk for lyophilization. The dosages of *L. plantarum* GKM3 freeze-dried powder in this study were adjusted to 5.1 × 10^9^ cfu/kg B.W./day as the high dose and 1.0 × 10^9^ cfu/kg B.W./day as the low dose for the mouse model by gavage. 

The senescence-accelerated prone mice P8 (SAMP8) were introduced in this study as an aging animal model. All SAMP8 mice were applied and kept at 12 h dark/light cycle under 25 ± 2 °C, 65 ± 5% RH with food and water ad libitum. The following experimental procedures were approved by the Animal Management Committee of the Department of Food and Nutrition, Providence University (Taichung City, Taiwan), with the IACUC number 20170629-A02.

### 2.2. Survival Test

In the survival test, six-month-old SAMP8 mice were grouped with 20 females and 20 males as follows: control, GKM3 high-dose group, and GKM3 low-dose group. Probiotic *L. plantarum* GKM3 was daily supplied to the treatment mice until they reached their natural death. The live/dead SAMP8 mice in each group were recorded and the average lifespans were calculated.

### 2.3. Experiment Design for Age-Related Cognitive Impairment

Three-month-old SAMP8 mice were housed for the cognitive impairment experiment. Both male and female SAMP8 mice were grouped as follows (n = 10 in each gender): control group, GKM3 high-dose group, and GKM3 low-dose group. The mice in the treatment groups were orally administered *L. plantarum* GKM3 for 14 weeks. The locomotion activity was conducted on the 11th week of the experiment. The passive avoidance test and active avoidance test were respectively conducted on the 13th and 14th week, and then mice were sacrificed for biochemical analysis and histological examination.

### 2.4. Locomotion Activity

Each mouse was placed in the center of a shuttle cage (25 cm × 25 cm × 25 cm, Coulbourn Instruments Model E61-21, Lehigh, PA, USA) and their locomotions were recorded by an activity monitor video-path analyzer for 10 min. The average time of mouse movement was analyzed every 5 min (s/5 min). This procession was conducted under dim light and silent conditions.

### 2.5. Passive Avoidance Test 

A shuttle cage (35 cm × 17 cm × 20 cm, Coulbourn Instruments Model E10-15, Lehigh, PA) was parted into a dark and light room. There was a guillotine door (7.5 cm × 6.5 cm, Coulbourn Instruments Model E10-15GD, Lehigh, PA, USA) between the two rooms. In the training test for the passive avoidance test, a mouse was placed in the light room for a minute adaptation and then the guillotine door was opened. The mouse would receive 0.5 μA electric shock for 0.5 s when it entered the dark room. The shock continually occurred three times every five seconds. The mouse was replaced under the same operation at 24 h, 48 h, and 72 h after the training session for the memory test, except that no shock was delivered. The average times of mice staying in the light room were measured. An upper cutoff time of 180 s was set. 

### 2.6. Active Avoidance Test

In the training test for the active avoidance test, the mouse would receive a 10 s conditioned stimulus (CS) based on light and sound, and then 5 s of 0.3 mA electric shock (unconditional stimulus, UCS) if the mouse stayed in the same room, whereas, there was no shock reception if the mouse moved to the other room. Each mouse had trained for 5 CS and UCS cycles. The mouse was tested under the same operation (CS/UCS system) with 15–20 min intertrial intervals four times a day and continually tested for four days. The average avoidance time was measured as the effect of learning memory.

### 2.7. Oxidative Stress Analysis

For the detection of 8-hydroxy-2′-deoxyguanosine (8-OHdG) from the brain mitochondrial DNA, 30 mg of brain tissue was extracted by the Blood & Tissue Genomic DNA Extraction Miniprep System (Viogene Biotek Corp., Taipei, Taiwan) and the highly sensitive 8-OHdG Check ELISA Kit (Nikken SEIL Co. Ltd., Shizuoka, Japan) was applied with an absorbance at 450 nm. For the thiobarbituric acid-reactive substances (TBARS) analysis, 25 mg of brain tissue was ground with 250 μL RIPA buffer and centrifuged at 16,000× *g* for 10 min under 4 °C. In total, 100 μL of supernatant was collected and added with 100 μL SDS solution and 4 mL color reagent, then boiled for an hour before the cooling treatment. Treated samples were centrifuged at 16,000× *g* for 10 min and placed for 30 min at room temperature. The TBARS content (mM/g protein) from each 150 μL sample was measured at 535 nm absorbance with the standard curve.

### 2.8. Measurements of the Brain Amyloid-β Protein

The amyloid-β (Aβ) protein from the brain tissue section was stained by the method of immunohistochemistry. The percentage of Aβ accumulation was analyzed by image system (Leica, Q500, Wetzlar, Germary) with a microscope under a 40× field. The calculation was based on the percentage of the Aβ accumulation area in all of the brain: (β-amyloid deposition area/whole brain area) × 100%.

### 2.9. Histology

The brains were excised and fixed with 10% formalin. The hippocampal tissue was processed through the process of dehydration, cleaning, infiltration, and embedding for sectioning. Then, the tissue of the hippocampus area was examined by Hematoxylin and Eosin (H&E) staining.

### 2.10. Statistic

The statistic stool SPSS 19.0 was used in this study. All data are expressed as mean ± S.E.M. and evaluated by one-way ANOVA for the comparison of means between the different groups. A significant difference was considered when the *p* value was less than 0.05 by Duncan’s multiple range tests. In the survival test, the Kaplan–Meier test was applied for analysis and the log-rank test was conducted to verify the differences between groups.

## 3. Results

### 3.1. Effect of L. plantarum GKM3 on the SAMP8 Survival Test

In the survival experiment, the lifespan of SAMP8 mice was observed until their natural death and it took 21 months to complete all the individual records. The average lifespan of non-treated male SAMP8 mice were 11.90 ± 0.18 months and for females it was 11.20 ± 0.21 months. In the probiotic GKM3 feeding groups, the average male lifespan extended to 12.05 ± 0.15 months and 13.50 ± 0.14 months in low and high doses of GKM3 treatments, respectively. The dose-dependent effect of probiotic GKM3 on lifespan extension in females could also be observed with 12.15 ± 0.21 months in the low-dose group and with 14.30 ± 0.17 months in the high-dose treatment (data not shown).

**Figure 1:**
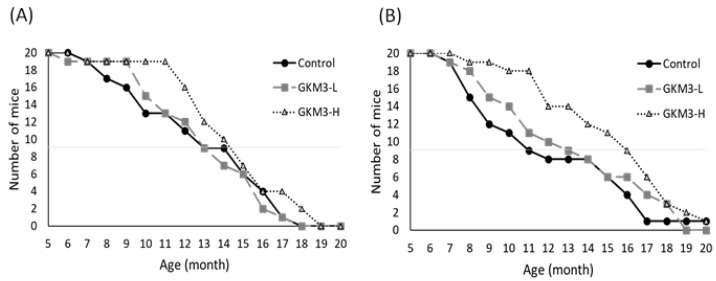
The lifespan of SAMP8 mice in males (**A**) and females (**B**). GKM3-L and GKM3-H were represent the groups fed probiotic GKM3 at the low dosage of 1.0 × 10^9^ CFU/kg BW/day and the high dosage of 5.1 × 10^9^ CFU/kg BW/day, respectively (n = 10).

To summarize life expectancy, the survival rate was estimated. With a 50% survival rate, the low dosage of GKM3 in the male group was 12.67 months and the high dosage of the GKM3 male group was 13.33 months, while the male control group only presented 12.50 months (Figure 1A). Similar to the observation in female SAMP8 mice, 12.00 months and 15.50 months with a 50% survival rate was showed in groups fed probiotic GKM3 at a dosage of 1.0 × 10^9^ CFU/kg BW/day and 5.1 × 10^9^ CFU/kg BW/day, respectively; however, 10.50 months with a 50% survival rate was observed in the female control group (Figure 1B). The female SAMP8 mice presented shorter life expectancies than males as the basis in this experiment; interestedly, the effect of *L. plantarum* GKM3 in terms of the lifespan had a greater impact on females. It is suggested that *L. plantarum* GKM3 can increase longevity in SAMP8 mice of both sexes, especially in female species. 

### 3.2. Body Weight, Food Intake, and Water Intake in SAMP8 Mice

In the experiment of aged-related cognitive impairment, there were no significant differences in the energy consumption and weight change among all the SAMP8 mice of the different treatment groups before the sacrifice (Table 1). This means that the probiotic GKM3 did not affect these metabolic parameters in the aged-mouse model. 

### 3.3. Effect of L. plantarum GKM3 on Memory Retention and Learning Enhancement

A locomotion activity test showed that there was no significant difference among all the SAMP8 mice in the first 5-min interval, regardless of gender (Table 2). Although there was a decrease in the second 5-min interval, when compared to the first 5-min interval, there was no significant change between the treated and non-treated groups.

**Figure 2:**
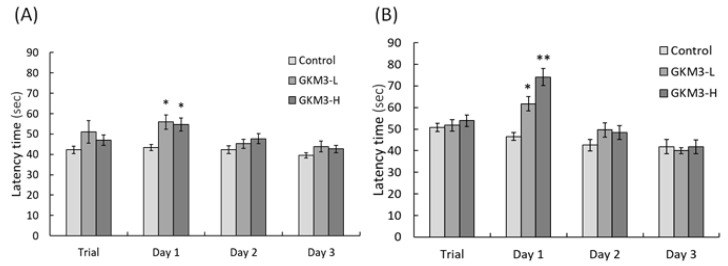
Passive avoidance ability of male (**A**) and female (**B**) SAMP8 mice. Values are presented as mean ± S.E.M. (n = 10). * *p* < 0.05 and ** *p* < 0.01 were regarded as a significant difference (one-way ANOVA). GKM3-L: 1.0 × 10^9^ CFU/kg BW/day (low dose); GKM3-H: 5.1 × 10^9^ CFU/kg BW/day (high dose).

Passive avoidance tests were performed on male and female groups of SAMP8 mice (Figure 2). There were no significant differences among all the groups during the trial test (*p* > 0.05). The latency of SAMP8 male mice fed with probiotic GKM3 was significantly increased in a comparison to the control group on the first day (*p* < 0.05) (Figure 2A). The time of female SAMP8 mice staying in the light room was also increased on the first day in the GKM3 treatment, with a *p*-value of less than 0.05 in the low-dose group and a *p*-value of less than 0.01 in the high-dose group when compared to the control. However, there were no significant differences after the second day of the passive avoidance test. A longer latency performed in the GKM3 feeding group, both in male and female SAMP8 mice, indicated that probiotic GKM3 could contribute to age-related memory retention and learning enhancement.

**Figure 3:**
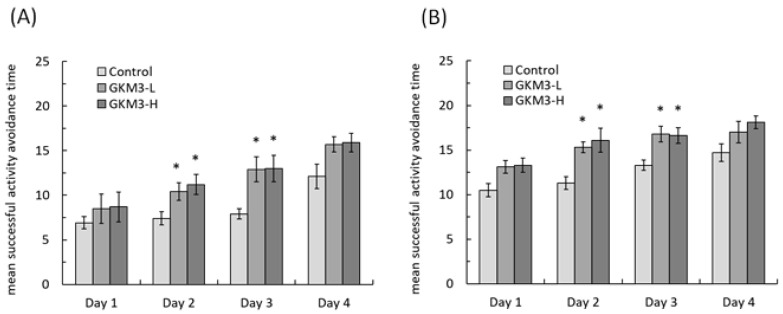
Active avoidance ability of male (**A**) and female (**B**) SAMP8 mice. Values are presented as mean ± S.E.M. (n = 10). * *p* <0.05 was regarded as a significant difference (one-way ANOVA). GKM3-L: 1.0 × 10^9^ CFU/kg BW/day (low dose); GKM3-H: 5.1 × 10^9^ CFU/kg BW/day (high dose).

In an active avoidance procedure, the mice were required to take any action to avoid the noxious stimulus. In the trial day of the active avoidance test, both the male and female SAMP8 mice fed with GKM3 showed a tendency of higher success in avoidance (Figure 3) but did not reach the statistic difference (*p* > 0.05) when compared with the control group. However, the result on day one and two of the active avoidance test showed that the successful time was significantly increased in the male groups with GKM3 fed when compared to the non-treated male SAMP8 mice (Figure 3A) (*p* < 0.05). A similar result was also observed in the female groups (Figure 3B). Interestedly, there were no statistical differences (*p* = 0.157) on the fourth day’s result between the control and probiotic groups. This means that the control mice needed training for four days to catch up with the presence of the probiotic-fed mice. It is suggested that the addition of probiotic GKM3 to the diet could help the host’s cognition which impacts the appropriate behavior in both genders.

### 3.4. Effect of L. plantarum GKM3 on Brain Oxidative Stress Reduction

**Figure 4:**
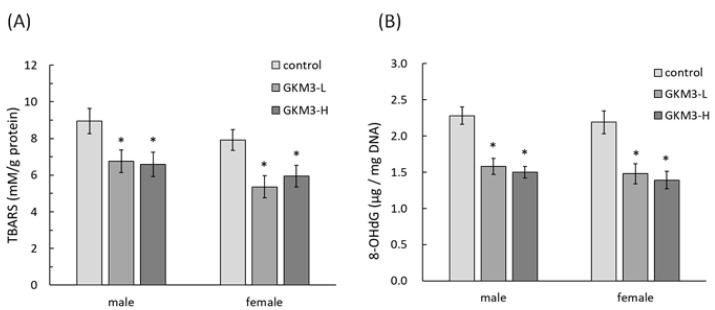
The TBARS (**A**) and 8-OHdG (**B**) level in SAMP8 mice brains after 14 weeks of probiotic GKM3 consumption. Values are presented as mean ± S.E.M. (n = 5). * *p* < 0.05 was regarded as a significant difference (one-way ANOVA). GKM3-L: 1.0 × 10^9^ CFU/kg BW/day (low dose); GKM3-H: 5.1 × 10^9^ CFU/kg BW/day (high dose). Abbreviations: TBARS, thiobarbituric acid reactive substances and 8-OHdG, 8-hydroxy-2′-deoxyguanosine.

TBARS is a by-product of lipid peroxidation which could describe the level of oxidative stress. The concentration of TBARS was detected in SAMP8 mice brains after the sacrifice. Both male and female SAMP8 mice fed with probiotic GKM3 showed a significant reduction in the concentration of brain TBARS when compared to the control mice (Figure 4A). Another marker of oxidative stress, 8-OHdG, in the brain was also significantly lower in the GKM3 treatment group in a comparison to the non-treatment (Figure 4B). This indicates that less DNA damage occurred in the probiotic group. There were no dose-effects of GKM3 observed in brain TBARS and 8-OHdG levels in either both genders, suggesting the low dosage of probiotic GKM3, which was 1.0 × 10^9^ CFU/kg BW/day, was enough for the antioxidant defense in aged mice.

### 3.5. Effect of L. plantarum GKM3 on Amyloid-β Participation in SAMP8 Mice Brains

**Figure 5:**
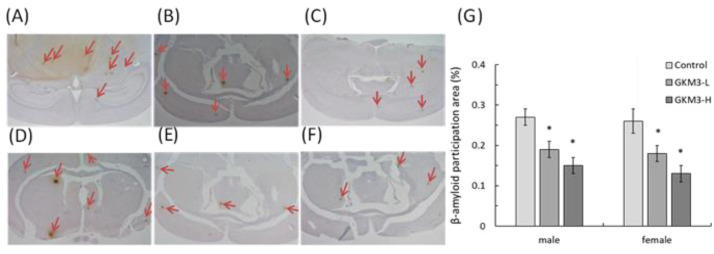
The β-amyloid participation in SAMP8 mice brains (40X) after 14 weeks of probiotic GKM3 consumption. (**A**): control male; (**B**): GKM3 low-dose male; (**C**): GKM3 high-dose male; (**D**): control female; (**E**): GKM3 low-dose female; (**F**): GKM3 high-dose female; and (**G**): the percentage of the amyloid-β participation area. The arrows point to the participation of amyloid-β. Values are presented as mean ± S.E.M. (n = 5). * *p* < 0.05 was regarded as a significant difference (one-way ANOVA).

Amyloid-β (Aβ) precipitation is one of the characteristics in SAMP8 mice which shared the same features with dementia at the clinical observation. Figure 5 shows the immunohistochemical result of the SAMP8 brain tissue. The Aβ precipitation was obviously observed in control male and female SAMP8 mice brains (Figure 5A,D), but little protein appeared on the samples of those treated with a low-dose of GKM3 (Figure 5B,E) and even less protein accumulated on those with the treatments with a high dose of GKM3 (Figure 5C,F). The percentage of the Aβ precipitation area in SAMP8 mice fed with probiotic GKM3 presented a significant reduction when compared to the controls in both genders (Figure 5G). This evidence revealed that *L. plantarum* GKM3 could prevent the age-related Aβ precipitation, which may contribute to a neurological disorder.

### 3.6. Effect of L. plantarum GKM3 on Hippocampus Histology in SAMP8 Mice

**Figure 6:**
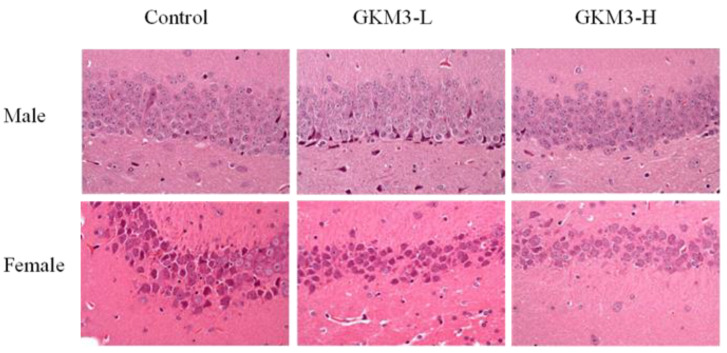
The hematoxylin and eosin staining of the hippocampus area in SAMP8 mice brains after 14 weeks of probiotic GKM3 consumption. GKM3-L: 1.0 × 10^9^ CFU/kg BW/day (low dose); GKM3-H: 5.1 × 10^9^ CFU/kg BW/day (high dose).

The hippocampus of SAMP8 mice brains was analyzed by hematoxylin and eosin staining (Figure 6). There were hyperchromic staining with the shrinking of nerve cells presented on the control and GKM3 low-dose groups. However, the neuron in the hippocampus of SAMP8 fed with a high-dose of GKM3 showed a tight arrangement. In addition, there was no abnormal observation of the cell structure and morphology in the GKM3 high-dose group. This indicates that probiotic GKM3 could delay the neuron damage caused by aging in the hippocampus of mice brains.

## 4. Discussion

There were no significant difference in the body weight or food intake between the control group and the probiotic groups (Table 1). This indicates that the probiotic GKM3 is safe and non-toxic to the mammal. Under the same metabolic parameter and energy consumption, the effects of anti-aging in GKM3-fed SAMP8 can be discussed in the following section.

Through observation of the lifespan of SAMP8, we note the death started from 6–7 months; however, both males and females in the probiotic GKM3-H group tended to present low death rates (Figure 1). Especially up to 11 months, the survival rates were very different. The GKM3-H group still kept 90–95% of the survival rate but the control group only presented 60–40% of the survival rate. Compared with other reported nutrients such as plant extract or marine sources on anti-aging effect, probiotic GKM3 provided a better result especially in terms of a longer survival rate [32,33]. It is possible that those anti-aging materials contained lipid, organic acid, polyphenols, or vitamins, which also can be easily found from the probiotic’s fermentation, and resulted in a longer lifespan [34,35,36].

Behavior avoidance tests are usually used for evaluating learning and memory in subjects. Memory is defined as a behavioral change caused by an experience, while learning is defined as a process for acquiring memory [37]. Both memory and learning were formed or achieved by being involved with nerve transmission and the nerve cell. Hence, the process of aging could increase the accumulation of ROS in neurons and damage the cell, resulting in poor memory and learning ability [38]. The passive avoidance test is a fear-motivated test in which subjects are required to inhibit a previously exhibited response [39]. Mouse with a better memory and learning ability can avoid entering dangerous areas. Conversely, active avoidance requires subjects to emit a response such as running to a safe place to avoid a noxious stimuli. Good memory and learning skills help the mice respond to alerting events and avoid incoming dangers. Evidence from our study showed that probiotic GKM3 could contribute to learning and memory by inhibiting the occurrence of undesirable responses, while there was no contribution in the control group even after the training procedure (Figure 2). Interestingly, the control group showed an increased tendency in the successful avoidance followed by the training days in the active avoidance test; however, the group fed with probiotic GKM3 showed a stronger significant successful avoidance (Figure 3). This could be explained by the trial-designed base [40]. Mice were forced to learn what to do in the active avoidance test but were relatively unstimulated in being required to learn what not to do in the passive avoidance test. In spite of the different reactions presented by the control mice, the effect of probiotic GKM3 in improving learning and memory could not be denied with the stimulations of both different mechanisms [41].

Similar behavior results were reported by Yong et al. with chicken extract as a diet addition and Su et al. with the supplementation of yam for SAMP8 mice [40,41]. As the bioactive compounds should exhibit a tremendous difference between the meat extract and plant origin, it could be speculated that the effect of cognitive maintenance was highly related to the alteration of gut microbiota [42,43]. Probiotics can produce metabolic compounds that enhance or suppress the growth of certain gut microorganisms [44]. These metabolic compounds, such as peptides, cortisol, or SCFAs, can also modulate the nervous system and maintain brain functions through the gut-microbiome-brain interaction. It was found that the gut microbiota in centenarians was very different from the aging population [45]. In particular, the relative abundance of *Firmicutes* was found. Even though we did not analyze the microbiota in this study, several papers pointed out that the administration of probiotics altered the gut microbiota [46,47,48,49,50]. In addition, our unrevealed data concerning a probiotic mixture maily contained *L. plantarum* GKM3 in a clinical trial that showed an increase of several *Bifidobacterium* species and several *Lactobacillus* species in the stool analysis after four weeks of consumption. It is suggested that *L. plantarum* GKM3 may maintain brain functions by changing the composition of gut microbial flora [51].

SAMP8 is a neuropathological model of accelerated brain aging derived from an AKR/J breeding colony by Professor Toshio Takeda at the Kyoto University [52]. Regarding age-associated morphological alteration, early amyloid accumulation in the hippocampus was found in SAMP8 mice, which resulted in learning disturbances and impaired memory [53]. Aβ was considered to induce ROS formation, lipid peroxidation, and neurotoxicity in hippocampal neurons [54]. Our results reveal that probiotic GKM3 not only inhibited oxidative stress in the brain (Figure 4) but also was involved in the upper inhibition of the amyloid formation (Figure 5). TBARS is formed as a by-product of lipid peroxidation and MDA is formed as its end-product. 8-OHdG is a common end-product of deoxyribonucleic acid (DNA) oxidation. That is, high levels of TBARS and 8-OHdG both represent strong oxidation and result in cognitive impairment [55].

Pyramidal cells, a type of populous neuron involved with the sensory and motor cues in the hippocampus, could contribute to information processing, learning, and memory [56]. The disordered arrangements of pyramidal cells in the hippocampal CA1 region were found in Alzheimer’s disease-affected mice [57]. It is indicated that *L. plantarum* GKM3 could alleviate the functional decline of neuron-transmitting by maintaining cell morphology (Figure 6). That is, long-term administration of probiotic GKM3 could enhance a better consciousness and encourage appropriate actions in life.

## 5. Conclusions

In this study, we examined the dose-dependent effect of long-term administration of *L. plantarum* GKM3 on longevity in both male and female SAMP8 mice. In addition, supplementation of probiotic GKM3 showed the improvement of memory and learning ability by being involved in anti-oxidative stress, by lowering Aβ accumulation, and by maintaining the arrangement of nerve cells in the hippocampus. These results suggest that probiotic *L. plantarum* GKM3 could act as an antioxidant for delaying the aging process and prevent age-related cognitive impairment. With its desirable functions and safe consumption history, *L. plantarum* GKM3 is a promising probiotic supplementation for the elderly.

## Figures and Tables

**Table 1:** Body weight, food intake, and water consumption.

Sex	Group	Body Weight (g)			Food Intake (g/day)	Water Consumption (mL/day)
		Initial (Week 0)	Final (Week 14th)	Gain		
Male	Control	28.37 ± 1.03 ^a^	32.06 ± 1.05 ^b^	3.69 ± 0.47 ^c^	4.79 ± 0.06 ^d^	6.67 ± 0.10 ^e^
	GKM3-L	28.81 ± 0.78 ^a^	31.14 ± 1.01 ^b^	2.33 ± 0.36 ^c^	4.74 ± 0.07 ^d^	6.65 ± 0.16 ^e^
	GKM3-H	28.58 ± 0.76 ^a^	32.12 ± 1.03 ^b^	3.55 ± 0.57 ^c^	4.71 ± 0.08 ^d^	6.53 ± 0.15 ^e^
Female	Control	27.14 ± 0.95 ^f^	27.61 ± 0.87 ^g^	0.47 ± 0.61 ^h^	4.09 ± 0.04 ^i^	4.64 ± 0.14 ^j^
	GKM3-L	27.01 ± 0.73 ^f^	28.05 ± 0.85 ^g^	1.49 ± 0.70 ^h^	3.98 ± 0.11 ^i^	4.60 ± 0.06 ^j^
	GKM3-H	26.63 ± 0.39 ^f^	28.51 ± 0.84 ^g^	1.88 ± 1.02 ^h^	4.10 ± 0.14 ^i^	4.69 ± 0.07 ^j^

Values are presented as mean ± S.E.M., n = 10 (one-way ANOVA). Same alphabet letters represent no significant difference. GKM3-L: 1.0 × 10^9^ CFU/kg BW/day (low dose); GKM3-H: 5.1 × 10^9^ CFU/kg BW/day (high dose).

**Table 2:** The locomotion activity of SAMP8 mice.

Sex	Group	Locomotion Time Interval (Minutes)^2^	
		0–5 (s/5 min)	6–10 (s/5 min)
Male	Control	94.90 ± 7.91 ^a^	86.40 ± 5.89 ^a^
	GKM3-L	111.10 ± 4.95 ^a^	84.80 ± 6.36 ^a^
	GKM3-H	92.70 ± 2.93 ^a^	80.20 ± 3.74 ^a^
Female	Control	85.10 ± 8.24 ^a^	84.10 ± 6.12 ^a^
	GKM3-L	88.10 ± 4.79 ^a^	72.10 ± 6.93 ^a^
	GKM3-H	121.20 ± 16.25 ^a^	117.70 ± 6.93 ^a^

Values are presented as mean ± S.E.M., n = 10 (one-way ANOVA). Same alphabet letters represent no significant difference. GKM3-L: 1.0 × 10^9^ CFU/kg BW/day (low dose); GKM3-H: 5.1 × 10^9^ CFU/kg BW/day (high dose).

## Data Availability

All data can be assessed from W.S. Lin via the email address.
